# Mortality Classification for Deaths With Nonfirearm Force by Police, 2012-2021

**DOI:** 10.1001/jamanetworkopen.2025.2371

**Published:** 2025-03-28

**Authors:** Justin Michael Feldman, Tracey Lloyd, Phillip Atiba Solomon

**Affiliations:** 1 Center for Policing Equity, West Hollywood, California; 2 François-Xavier Bagnoud Center for Health and Human Rights, Harvard University, Boston, Massachusetts; 3 Department of African American Studies, Yale University, New Haven, Connecticut; 4 Department of Psychology, Yale University, New Haven, Connecticut

## Abstract

**Importance:**

For deaths preceded by nonfirearm force by law enforcement officers, mortality classification has potential implications for public accountability and epidemiologic surveillance.

**Objective:**

To characterize the proportion of in-custody deaths for which cause and manner of death reflected the use of nonfirearm force by police officers and to assess factors associated with mortality classification.

**Design, Setting, and Participants:**

This cross-sectional study examined national data from the Associated Press’s Lethal Restraint database. The analysis included deaths that occurred in 2012 to 2021 following the use of nonfirearm force by state or local police officers and that did not occur in prisons or jails.

**Exposures:**

Death investigation system (medical examiner, coroner, or sheriff-coroner), racial and ethnic bias, and county political context (percentage of Republican presidential votes).

**Main Outcomes and Measures:**

Manner of death classification (homicide vs accident, undetermined, natural, suicide, and any manner), mention of force-related injuries or conditions in the cause-of-death statement (yes vs no), and mention of any force in the cause-of-death statement (yes vs no) were assessed using logistic regression models.

**Results:**

A total of 940 decedents (mean [SD] age, 39 [11] years; 909 men [97.0%]; 297 identified as Black [32.4%], 179 as Hispanic or Latinx [19.6%], 401 as White [43.9%], and 37 as other [4.0%] race and ethnicity) were included. Of the 940 deaths, 268 (28.5%) were classified as homicide, and 155 (16.5%) were mentioned as a force-related injury or condition and 400 (42.6%) as any force in the cause-of-death statement. In contrast, 695 cause-of-death statements (73.9%) mentioned drugs. Unadjusted results showed that homicide classification increased from 25.0% (66 of 264 deaths) during 2012-2014 to 32.2% (123 of 382 deaths) during 2018-2021. Models estimating adjusted prevalence differences showed that compared with medical examiner jurisdictions, coroners (−0.19; 95% CI, −0.31 to −0.06) and sheriff-coroners (−0.17; 95% CI, −0.28 to −0.05) were less likely to classify deaths as homicides. Model results also showed that classifications for incidents occurring in counties with the lowest percentage of Republican voters were the most likely to reflect force across all 3 manner and cause outcomes (0.17 [95% CI, 0.05-0.28] for homicide, 0.22 [95% CI, 0.06-0.38] for any mention of force, and 0.14 [95% CI, 0.023-0.26] for force-related injuries or conditions).

**Conclusions and Relevance:**

In this cross-sectional study of deaths following use of nonfirearm force by police officers, nonhomicide classifications and cause-of-death statements making no mention of force were widespread. These findings suggest that inconsistent classification of the cause and manner of deaths that follow nonfirearm force by police officers is an issue of public safety and health with profound social implications.

## Key Points

**Question:**

How do coroners and medical examiners categorize in-custody deaths that follow use of nonfirearm force by US law enforcement officers?

**Findings:**

In this cross-sectional study of 940 in-custody deaths, 28.5% were classified as homicides and 42.6% of cause-of-death statements mentioned any force, while 73.9% of cause-of-death statements mentioned drugs. Deaths investigated by coroners and sheriff-coroners and occurring in Republican-leaning counties were less likely to have use of force reflected in cause-of-death statements.

**Meaning:**

These findings suggest that most categorizations used for US in-custody deaths do not identify the potentially causal role of police use of force, which may have implications for public policy and public health surveillance.

## Teaser

This cross-sectional study characterizes the proportion of in-custody deaths for which cause and manner of death reflected the use of nonfirearm force by police officers and assesses factors associated with mortality classification.

## Introduction

In recent years, US medical and public health professional associations have called for improved data collection and investigation for police-related deaths,^1,2^ which are often misclassified in mortality data.^3^ Prior public health research on deaths that follow police officer use of force often focused on the approximately 90% of these fatal incidents resulting from police shootings.^3^ For deaths that follow nonfirearm force (eg, chokeholds, prone restraint, conducted energy weapon shocks), mortality classification presents unique challenges. These deaths are especially likely to be classified in ways that are inconsistent and do not reflect the violent confrontation that preceded the death.^3,4,5^ Mortality classification has potential ramifications at the level of an individual death (eg, in legal proceedings) and systemically (eg, for monitoring social inequities or the safety of restraint practices).^6^ However, previous research on classification of deaths following nonfirearm force has been limited to journalistic accounts, opinion surveys of forensic pathologists, and analyses of small samples of mortality data.^3,4,5,7^

In the US, medical examiners (appointed officials, typically forensic pathologists) and coroners (generally, elected officials without medical training) investigate deaths that occur during police encounters and make determinations about the manner and cause of death. Manner of death is a classification based on the circumstances of a death and includes natural, accident, suicide, homicide, and undetermined.^8^ Homicide is defined by the forensic pathology community as death at the hands of another and does not necessarily imply an unlawful act. The National Association of Medical Examiners, the primary US professional association for forensic pathologists, suggests classifying deaths resulting from police restraint or subdual as homicide,^1^ but surveys have indicated that death investigators routinely select other manners of death when presented with a description of a typical death that occurred during police restraint.^5^ In contrast, cause of death is a written statement of the diseases, injuries, or complications that directly caused or contributed to a death. Cause-of-death statements for high-profile incidents have included force-related injuries or conditions (eg, neck compression in George Floyd’s cause of death),^9^ but these cases may be exceptional. Prior evidence has suggested that a large proportion of cause-of-death statements following nonfirearm force solely identify cardiovascular conditions, drugs, and/or psychiatric illness as causes.^3^

Death investigators have a high degree of discretion in mortality classification decisions and may assign manner and cause in any combination. Their determinations have consequences for the inclusion and characterization of deaths that appear in public health and safety data systems used to monitor violence-related mortality.^6^ While deaths involving impact weapons (eg, batons) or strikes by an officer’s body (eg, punches, kicks) may result in traumatic injury identifiable on autopsy, those following restraint (eg, conducted energy weapon shocks, prone restraint) may yield no anatomic findings that point to a functional cause of death.^10^ For restraint deaths, investigators may draw on competing explanations for restraint deaths in forensic pathology literature, such as positional asphyxia or, more recently, prone-restraint cardiac arrest,^11^ which posit that restraint may cause death by limiting respiration (or inducing dysrhythmias by conducted energy weapon shocks). Other explanations, such as the highly contested excited delirium or agitated delirium (characterized by hyperactive, agitated behavior and often related to drug intoxication), posit that the role of restraint in the deaths is incidental.^12^

Political pressure and conflicts of interest may also play a role in the death investigation process.^13^ In some jurisdictions, the sheriff serves as the coroner and may be charged with investigating their own agency’s actions.^14^ Furthermore, research has suggested that anti-Black racial bias may influence the classification of injury-related deaths, including those occurring in jails, although to our knowledge, no study has examined racial and ethnic bias for the classification of deaths in police custody.^4,15^ Finally, local political environments may influence death classifications. Prior research has suggested that COVID-19 deaths were more likely to be misclassified in areas with stronger support for the Republican Party.^16^ Deaths following use of force are also highly politicized in the US, and classification may reflect higher perceptions of police legitimacy reported among Republicans vs Democrats.^17^

Our study is, to our knowledge, the first national analysis of mortality classification for deaths that followed nonfirearm force by US police officers. We leveraged the Associated Press’s Lethal Restraint database, a national dataset published in April 2024 that contains details on 1036 nonfirearm deaths that occurred in the US during 2012-2021 and did not involve force used in a jail or prison.^18^ Types of force preceding the enumerated deaths include those typically characterized as restraint (eg, hogtying, conducted energy weapon shocks), as well as other nonfirearm force types (eg, baton or fist strikes, canine bites). The Associated Press has noted that the dataset includes approximately 270 deaths not previously reported in existing open-source databases.^19^

The aim of our study was to quantify the proportion of these incidents classified as resulting from police officer use of force and, therefore, would be characterized as violence-related deaths in public health data systems. We hypothesized that cause-and-manner classifications would more likely reflect use of force in medical examiner jurisdictions (vs coroner and sheriff-coroner jurisdictions), in counties with low Republican Party voting concentrations (vs those with high concentrations), and for White (vs African American or Black) decedents.

## Methods

This cross-sectional study analyzed solely public-use data and no data on living persons; thus, local ethics review and informed consent were not required in accordance with the Common Rule. This study followed the Strengthening the Reporting of Observational Studies in Epidemiology (STROBE) reporting guideline for cross-sectional studies.

We classified death investigation systems and added county-level variables based on incident locations for each death in the Lethal Restraint database. To construct the Lethal Restraint database, journalists conducted an open-source search of deaths that followed the use of nonfirearm force by law enforcement officers that occurred in the 50 states and District of Columbia during 2012-2021. They excluded incidents that occurred in jails and prisons, solely involved federal agents, or involved handcuffs as the only force. The Associated Press did not make judgments about whether force caused death; deaths were temporally associated with force, typically meaning that they occurred during the police encounter or hospital stay that immediately followed. Data collection included decedent demographics, force types, cause and manner of death, and the death investigation offices involved.

The Associated Press obtained data from official documents, personal communications with public officials, and videos when available. Cause and manner of death were most often obtained from autopsy reports (accounting for the source in approximately 60% of deaths) and included communications with death investigators and documents from state police investigations. Official death certificates were only available as a source document for less than 10% of deaths. We geocoded Lethal Restraint incident locations to counties, then added county poverty rates (5-year estimates from the American Community Survey), as well as urbanicity categories.^20^

We excluded deaths for which there was evidence of traumatic injury that was self-inflicted, caused by gunshots, or inflicted by a third party. Such injuries are atypical for deaths following nonfirearm force and may complicate mortality classification.

### Outcomes

To characterize whether a death’s classification reflected police officer use-of-force, we created 3 binary outcome categories based on the cause-of-death statement and manner of death (Table 1). The first category was death classified as a homicide. The second category was mention of specific force-related injuries or conditions in the cause-of-death statement, including traumatic injury (eg, fractures, wounds, blunt force trauma), asphyxia, compression (eg, of the neck or chest), or cardiac conditions related to conducted energy weapon shocks. We categorized the latter as a force-related condition only when the weapon was also mentioned (eg, conducted energy weapon–induced arrhythmias), as the same cardiac conditions may result from other chains of events, such as drug overdose. The third category was any mention of force in the cause-of-death statement (eg, restraint, conducted energy weapon shocks, neck holds). For this category, we interpreted force-related injuries or conditions as implying use of force, so for example, asphyxia would be considered a mention of force even if the restraint that caused the asphyxia was unmentioned. As a result, all deaths meeting the criteria for the second outcome category are also included in the third.

**Table 1.** Description of Death Categorization Terms

Term	Definition	Notes
Manner of death	Nosologic classification (on a death certificate or autopsy report) of circumstances that led to the death. Typically includes homicide, natural, suicide, accident, and undetermined.	Natural deaths are caused by disease, while homicide, accident, and suicide are due to injury. Coroners and medical examiners vary in the level of uncertainty required to select undetermined manner.
Homicide	A manner-of-death category denoting that the death occurred at the hands of another by means other than an accident.	Homicide does not imply an unlawful action. Debates exist about whether homicide should include volitional acts that were not intended to cause death. In the US, the National Association of Medical Examiners promotes homicide classification for deaths related to police restraint and subdual techniques.
Cause-of-death statement	Text field on a death certificate or autopsy report describing the chain of events, ie, diseases, injuries, or complications, that directly caused the death.^21^ We also included contributing causes, defined as “other significant conditions contributing to death but not resulting in the underlying cause.”	Evidence of drug intoxication may appear on a postmortem toxicologic analysis but not in the cause-of-death statement, suggesting that the pathologist determined that the substance did not cause the death.
Force-related injury or condition	For this study, defined as injuries or conditions resulting from police officer use of force as mentioned in the cause-of-death statement. These injuries or conditions may include traumatic injury, compression (eg, of the neck or chest), asphyxia, or arrhythmias induced by conducted energy weapon shocks. They do not include nonspecific mechanisms of death, such as cardiac arrest.	Example cause-of-death statements mentioning force-related injuries or conditions include blunt force head injury, asphyxia from active prone restraint (restraint asphyxia), and cardiac arrhythmia due to conducted electrical weapon discharge.
Force	For this study, defined as types of force used by police officers that are mentioned in the cause-of-death statement (eg, restraint, conducted energy weapon shocks, neck holds). We included mentions of force even when the attribution of the death to the force was unclear (eg, a death occurring in the setting of restraint). We also included any mention of a force-related injury or condition, eg, asphyxia, as this implies force.	Example cause-of-death statements mentioning force include cocaine toxicity and stress during physical restraint and methamphetamine intoxication complicating profound physical exertion and police restraint to include carotid sleeper hold.

We additionally categorized cause-of-death statements that mentioned drugs or excited/agitated delirium. For all categories, 2 analysts (J.M.F. and a nonauthor research assistant) reviewed the cause-of-death statements and reached consensus for cases in which categorization was initially discordant. Cause-of-death categorizations were also based on any available contributing cause statements.

### Exposures

We classified the medicolegal death investigation system (medical examiner, coroner, or sheriff-coroner) for each death based on the type of office that had legal jurisdiction over the death. Although the name of the agency that performed the autopsy was available in the Lethal Restraint database, in some cases, this was not the agency with legal jurisdiction over the death investigation due to outsourcing arrangements. We inferred legal jurisdiction and classified investigation systems by following a set of decision rules, which we present in the eAppendix in Supplement 1, along with a sensitivity analysis that accounts for the outsourcing of autopsies by coroners.

We measured the concentration of Republican Party voters based on the percentage of bipartisan votes that were for the Republican presidential candidate in the incident county (ie, Republican vote count divided by the Republican plus Democrat vote count). We calculated this variable using county-level data for 2012-2020,^22^ with the value based on the presidential election that occurred in the year of death or the most recent presidential election relative to the death year.

We used the Lethal Restraint database’s race and ethnicity data, which journalists derived from official documents, to examine racial and ethnic differences in classifications that may be attributable to bias. We classified non-Hispanic decedents as African American or Black (hereafter, Black), White, or other (American Indian or Alaska Native, Asian, Pacific Islander, or multiracial), while Hispanic and Latinx included individuals of any race.

### Statistical Analysis

In addition to descriptive tabulations, we fit separate logistic regression models for each of the 3 outcomes and 3 hypotheses, for a total of 9 outcome models. All models were complete case analyses. We weighted outcome models using inverse propensity scores (estimated using the WeightIt package in R, version 4.4.0 [R Foundation]), and fit continuous variables in outcome models using restricted cubic splines.^23^ Propensity scores were estimated as the inverse probability of exposure weights for each of the 3 exposures (bayesian additive regression trees as the method and average treatment effect on the treated as the estimand). All propensity and outcome models were adjusted for decedent age, force type, incident state, county urbanicity, county poverty, and year and month of death. All models also included (as covariates or as the dependent variable, each corresponding propensity model) investigation system, county percentage of Republican voters, and decedent race and ethnicity. After fitting the weighted logistic regression models with standard errors clustered by autopsy agency, we used the R marginaleffects package^24^ to estimate adjusted prevalence differences for each exposure-outcome combination. We determined results to be inconclusive when their 95% CI contained the null effect (ie, an adjusted prevalence difference of 0).

## Results

A total of 940 decedents were included (mean [SD] age, 39 [11] years; 909 men [97.0%] and 28 women [3.0%]; 297 identified as Black [32.4%], 179 as Hispanic or Latinx [19.6%], 401 as White [43.9%], and 37 as other [4.0%] race and ethnicity) (Table 2). Of the 1036 deaths included in the Lethal Restraint database, we excluded 11 listed as contributing causes of death (8 involving self-inflicted wounds, 1 involving wounds inflicted by another civilian, and 2 involving gunshot wounds to the limbs). We removed a further 85 deaths due to missing cause and/or manner data, yielding a sample of 940 deaths used for the descriptive tables. Cases with any missing exposure or covariate data (Table 2) accounted for less than 5% of the 940 deaths, and all models analyzed the 906 complete cases.

**Table 2.** Manner- and Cause-of-Death Statement Categorizations Stratified by Selected Variables (N = 940)

Characteristic	No. of deaths	Manner and cause of death, No. (%)^a^		
		Homicide (n = 268)	Force-related injury or condition (n = 155)	Any force (n = 400)
Decedent age (2 missing [0.2%])				
<30 y	179	47 (26.3)	33 (18.4)	71 (39.7)
30 to <45 y	489	136 (27.8)	73 (14.9)	202 (41.3)
≥45 y	270	85 (31.5)	49 (18.2)	125 (46.3)
Period (0 missing)				
2012-2014	264	66 (25.0)	52 (19.7)	113 (42.8)
2015-2017	294	79 (26.9)	44 (15.0)	122 (41.5)
2018-2021	382	123 (32.2)	59 (15.5)	165 (43.2)
Decedent race or ethnicity (26 missing [2.8%])				
African American or Black	297	71 (23.9)	46 (15.5)	116 (39.1)
Hispanic/Latinx	179	55 (30.7)	31 (17.3)	81 (45.3)
White	401	114 (28.4)	73 (18.2)	173 (43.1)
Other^b^	37	19 (51.4)	5 (13.5)	23 (62.2)
Decedent sex (3 missing [0.3%])				
Female	28	6 (21.4)	6 (21.4)	11 (39.3)
Male	909	261 (28.7)	149 (16.4)	387 (42.6)
County percentage of Republican voters (0 missing), quartile				
1 (Lowest)	240	79 (32.9)	45 (18.8)	117 (48.8)
2	246	82 (33.3)	40 (16.3)	115 (46.8)
3	233	59 (25.3)	43 (18.5)	100 (42.9)
4 (Highest)	221	48 (21.7)	27 (12.2)	68 (30.8)
Investigation system (0 missing)				
Medical examiner	603	196 (32.5)	105 (17.4)	279 (46.3)
Coroner	222	46 (20.72)	34 (15.3)	77 (34.7)
Sheriff-coroner	115	26 (22.61)	16 (13.9)	44 (38.3)

^a^ Percentages refer to the percentage of deaths within each stratum that are classified as homicide, mention of a force-related injury or condition, or mention of any force.

^b^ Includes American Indian or Alaska Native, Asian, Pacific Islander, or multiracial.

Of the 940 deaths, 268 (28.5%) were classified as homicide, and 155 (16.5%) and 400 (42.6%) were assigned cause-of-death statements mentioning a force-related injury or condition and any force, respectively (Table 3). Regarding manner of death, accident (441 deaths [46.9%]) was more common than homicide, while undetermined (183 deaths [19.5%]), natural (57 deaths [5.0%]), and suicide (1 death [0.1%]) were less common. Homicide determinations increased over time, rising from 25.0% of deaths (66 of 264) during 2012-2014 to 32.2% of deaths (123 of 382) during 2018-2021.

**Table 3.** Cause-of-Death Statement Categorizations by Manner of Death

Cause-of-death statement	Manner of death, No. (%)					
	Homicide (n = 268 [28.5%])^a^	Accident (n = 441 [46.9%])^a^	Undetermined (n = 183 [19.5%])^a^	Natural (n = 47 [5.0%])^a^	Suicide (n = 1 [0.1%])^a^	Any (N = 940 [100%])^a^
Force-related injury or condition mentioned	82 (30.6)	37 (8.4)	33 (18.0)	3 (6.4)	0	155 (16.5)
Any mention of force	200 (74.6)	83 (18.8)	111 (60.7)	6 (12.8)	0	400 (42.6)
No mention of force or force-related injury or condition	68 (25.4)	358 (81.2)	72 (39.3)	41 (87.2)	1 (100)	540 (57.5)
Drugs mentioned	168 (62.7)	401 (90.9)	119 (65.0)	6 (12.8)	1 (100)	695 (73.9)
Excited/agitated delirium mentioned	27 (10.1)	92 (20.9)	32 (17.5)	8 (17.0)	0	159 (16.9)

^a^ Percentages in brackets refer to total deaths by manner. Other percentages refer to deaths in particular manner-cause combination strata.

For the 940 cause-of-death statements, 159 (16.9%) mentioned excited/agitated delirium, and 695 (73.9%) mentioned drugs. A total of 593 deaths mentioned cocaine, amphetamines, and/or phencyclidine, comprising 89.0% of the 666 cause-of-death statements mentioning specific drugs. Compared with accident, deaths classified as homicide were more likely to mention any force (83 deaths [18.8%] for accident vs 200 deaths [74.6%] for homicide) and force-related injuries or conditions (37 deaths [8.4%] vs 82 deaths [30.6%]) and less likely to mention drugs (168 deaths [62.7%] vs 401 deaths [90.9%]) and excited/agitated delirium (27 deaths [10.1%] vs 92 deaths [20.9%]).

Model results suggested that coroners and sheriff-coroners were less likely to classify the manner of death as homicide compared with medical examiners (Figure). Adjusted prevalence differences (reference group: medical examiner jurisdictions) were −0.19 (95% CI, −0.31 to −0.06) for coroners and −0.17 (95% CI, −0.28 to −0.05) for sheriff-coroners. Differences in cause-of-death classification (force-related injuries or conditions and any mention of force) by investigation system were inconclusive.

**Figure.**
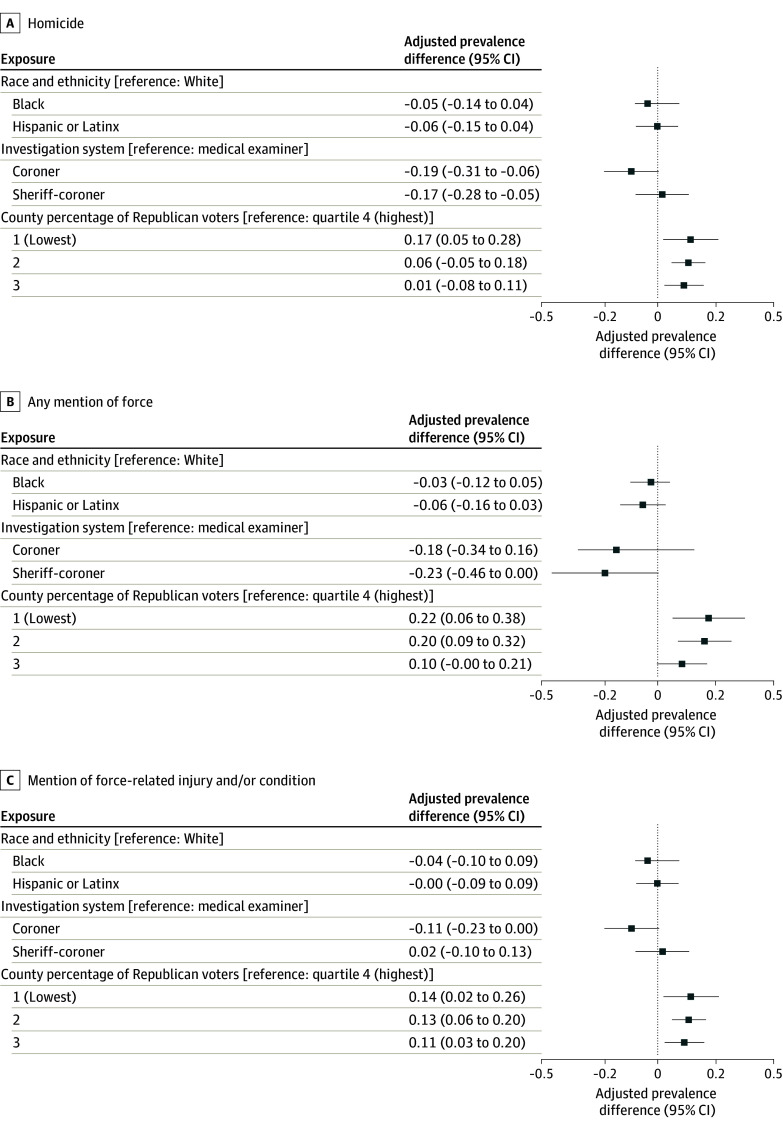
Adjusted Prevalence Difference Estimates for Factors Associated With Mortality Classification

Counties in the highest quartile of Republican vote share were consistently least likely to reflect force in cause-and-manner classifications (Figure). Model-adjusted prevalence differences (lowest vs highest Republican quartile) were 0.17 (95% CI, 0.05-0.28) for homicide, 0.22 (95% CI, 0.06-0.38) for mentions of force, and 0.14 (95% CI, 0.02-0.26) for force-related injuries or conditions.

Black persons comprised 32.4% of decedents vs 13.1% of the US population during the study period.^25^ A notably lower proportion of Black persons’ deaths were classified as homicide vs White persons’ deaths (71 [23.9%] vs 114 [28.4%], respectively), and Black decedents were also least likely to have mentions of any force or force-related injuries or conditions than White decedents (46 [15.5%] vs 73 [18.2%], respectively) (Table 2). However, model-adjusted results were inconclusive across all outcomes.

## Discussion

Our national cross-sectional study of deaths that followed nonfirearm force by US police officers found that mortality classification often did not mention or reflect the use of force. Classifying deaths as nonhomicide and writing cause-of-death statements that made no mention of force were widespread practices in places with all death investigation system types and political characteristics. While the National Association of Medical Examiners recommends assigning homicide as a manner of death resulting from police restraint or subdual, 71.5% of the incidents in the Lethal Restraint database included in our analytic dataset were categorized as nonhomicide, suggesting that death investigators either did not follow the recommendation or did not attribute these deaths to police officer actions. Death records that neither were classified as homicide nor made any mention of force comprised approximately one-half of the incidents in our dataset. Nonhomicide, nonforce determinations may hinder efforts to identify in-custody deaths in public health databases and may make it less likely for these deaths to receive public attention.

In recent years, US professional and legislative advocacy efforts have focused on banning or discouraging the use of the term excited delirium in cause-of-death statements for deaths during police encounters.^26,27^ However, we found that excited/agitated delirium appeared in fewer than 1 in 5 cause-of-death statements. More typically, nearly 3 in 4 statements cited drug toxicity. Excited delirium may not have been mentioned because the investigator did not believe that the condition caused the death. Furthermore, not mentioning excited delirium may reflect an investigator’s beliefs about nosologic classification. For example, if the investigator believed that the excited delirium was drug-induced, they may have found it more appropriate to cite drug toxicity alone in the cause-of-death statement. It should also be noted that US law enforcement and medical researchers have identified drug intoxication, particularly when involving stimulants or phencyclidine, as a risk factor for restraint-related deaths requiring reforms to restraint practices (eg, avoiding neck holds and prone positioning).^28,29^ While the physiologic mechanism is unclear, recent literature has proposed a pathway by which drug intoxication increases metabolic demand and, when restraint hinders the ability to exhale sufficiently, metabolic acidosis results in cardiac arrest.^11^

Deaths reported in the Lethal Restraint database were disproportionately concentrated among the Black decedents, for whom mortality classification was also least likely to reflect force. Although the study could not draw conclusions about whether racial and ethnic differences in classifications were attributable to bias, it is important to consider the influence of institutional racism on the forensic pathology community’s evaluation of police restraint safety. In arguing that deaths following restraint are primarily due to underlying disease states rather than restricted breathing, some researchers and pathologists have speculated that disproportionate restraint-related death rates among the Black population arise from the group’s higher prevalence of sickle cell trait, crack cocaine use, or excited delirium–inducing genotypes.^30,31,32^ These conjectures rely on biological and cultural essentialism, and their proponents have not considered an alternative explanation for racial inequality that challenges their view of restraint as incidental to mortality: Discriminatory policing exposes Black populations to higher levels of dangerous restraint practices.

Our finding that coroners and sheriff-coroners were less likely to classify deaths as homicides is in line with prior research suggesting that compared with medical examiners, sheriff-coroners may be less likely to report in-custody deaths to law enforcement or public health data systems.^14^ The relative autonomy provided to medical examiners through their role as appointed officials may, at least in part, insulate them from the political pressures that many death investigators report.^13^

The pathways by which Republican-leaning counties are least likely to classify deaths in ways that reflect nonfirearm force remains unclear. It is possible that the deaths are investigated less thoroughly in such counties perhaps because investigators have closer, more sympathetic relationships with police officers or may have less access to footage from body-worn cameras^33^ that may otherwise aid in death investigations.

### Limitations

Our study has several limitations. First, it was not possible to assess the completeness of the dataset given a lack of official US data on police-related deaths, and the data also exclude an unknown number of incidents involving federal law enforcement alone. Second, the cause and manner of death were most often obtained from autopsy reports, which are produced by forensic pathologists. In many coroner jurisdictions, the coroner has the authority to overrule pathologists. Individual pathologists have reported instances in which a coroner changed their homicide determination to accident for a police-related death.^34^ Finally, with few deaths investigated by each agency and no data on the specific pathologist, we were unable to conduct a racial and ethnic bias assessment that included within-agency or within-investigator comparisons.

## Conclusions

This cross-sectional study suggests that the inconsistent classification of the cause and manner of deaths that follow nonfirearm force by police officers is an issue of public safety and public health data quality that has profound social implications. Further research may assess interventions designed to improve reporting.

## Notes

Supplement 1.
eAppendix.

Supplement 2.
Data Sharing Statement
