# Supplementary material for: Mortality Classification for Deaths With Nonfirearm Force by Police, 2012-2021
Source: JAMA Netw Open. 2025 Mar 28;8(3):e252371. doi: 10.1001/jamanetworkopen.2025.2371 (PMC11953755; doi:10.1001/jamanetworkopen.2025.2371)
Supplement: Supplement 2. — Data Sharing Statement [file jamanetwopen-e252371-s002.pdf]

Data Sharing Statement

Feldman. Mortality Classification for Deaths With Non-Firearm Force by Police, 2012-2021. *JAMA Netw Open*. Published March 28, 2025. doi:10.1001/jamanetworkopen.2025.2371

Data
Data available: Yes
Data types: Data (not involving human participants)
How to access data: https://github.com/justinfeldman/lethal-restraint
When available: With publication

Supporting Documents
Document types: Statistical/analytic code
How to access documents: https://github.com/justinfeldman/lethal-restraint
When available: With publication

Additional Information
Who can access the data: Anyone requesting the data
Types of analyses: For any purpose
Mechanisms of data availability: All data and code are open-access and available at the URL provided
